# Response to Zoledronic Acid Infusion in Children With Fibrous Dysplasia

**DOI:** 10.3389/fped.2020.582316

**Published:** 2020-11-24

**Authors:** Sujit Kumar Tripathy, Shakti Swaroop, Sandeep Velagada, Debashree Priyadarshini, Rashmi Ranjan Das, Amit Kumar Satpathy, Kanhaiyalal Agrawal

**Affiliations:** All India Institute of Medical Sciences Bhubaneswar, Bhubaneswar, India

**Keywords:** bisphosphonates, zoledronic acid, cafe-au-lait spots, pediatric, adolescent, benign bone disease, lytic lesion

## Abstract

**Objective:** This retrospective study evaluated the outcome and safety of long-term treatment with zoledronic acid, in both polyostotic and mono-ostotic fibrous dysplasia (FD) of children.

**Methods:** The case records of children and adolescents with symptomatic FD who received zoledronic acid (0.1 mg/kg IV infusion over 1 h) and have completed at least 2 years follow-up were analyzed. The relevant details were recorded in a predesigned chart. Clinical assessment [pain assessment by visual analog scale (VAS) and incidence of new fracture], radiological changes (cortical thickening, ossification, and decrease in the diameter of the osteolytic lesions) and biochemical parameters [alkaline phosphatase (ALP)] were used to evaluate the improvement.

**Results:** The mean age of presentation was 9.1 years, with four males and six females. All patients had symptomatic FD in the lower limb with complaints of pain, tenderness, swelling, or deformity. Four children had associated pathological fracture. The radiological evaluation with bone scan revealed polyostotic involvement in eight patients and mono-ostotic involvement in two patients. Three patients had associated systemic features like café-au-lait spots or precocious puberty. The fracture united within 3 months and the radiological improvement was evident in the form of filling of the osteolytic defect. The pain score in six patients showed significant improvement (VAS < 3). The ALP level decreased to 544.12 ± 47.35 IU/L from an initial value of 895.75 ± 79.64 IU/L (*p* = 0.04) at 12 months. One patient had symptomatic hypocalcaemia after zoledronic acid infusion.

**Conclusion:** The clinical and radiological response of zoledronic acid treatment in FD of children is promising. Further randomized control trials with a larger sample size are required to establish this drug as a first-line medical treatment in FD.

## Introduction

Fibrous dysplasia (FD), also called as “Lichtenstein–Jaffe disease” is a benign developmental disorder of the bone affecting solitary bone or multiple bones (1). It accounts for 5–7% of the primary benign bone tumors (1). Mono-ostotic FD (MFD) comprises 70–80% of the cases. McCune Albright syndrome (MAS) is a rare syndrome where polyostotic FD (PFD) is associated with endocrine abnormalities and pigmentation of the skin (2). FD has a predilection for long bones, ribs, and craniofacial bones. FD can present with swelling, pain, deformity, or with pathological fractures (1, 3). More than half of the patients present before the third decade of life, and the PFD usually presents in the first decade of life (1–3). MFD presents between the ages of 5–20 years. Both males and females are equally affected. Many of the patients may not have any symptoms, and it may be an incidental finding (1, 3).

A missense mutation in the gene coding for the alpha subunit of the stimulatory G protein, Gs, in the GNAS complex locus in chromosome 20q13 leads to abnormal proliferation and differentiation of bone marrow stromal cells (4, 5). As a result, abnormal bone tissue is formed in the fibrous stroma, and there is increased osteoclastic activity. Increased production of interleukin 6 (IL-6) is also linked to increased osteoclastic activity locally (3–5). The increased osteoclast activity results in the destruction of normal bone, and there is excessive formation of fibrous tissue in the medulla with abnormal bone causing extensive tumor-like bone lesions. As there is no mature lamellar bone formation, it cannot remodel under stress and get deformed.

The treatment of FD varies because of heterogeneous clinical manifestations that range from asymptomatic lesions to severe painful lesions with fractures (6). As a result, analgesics, bisphosphonates, calcium, vitamin D, and surgical corrections (for pain relief, deformity correction, and stabilization of pathological fractures) are employed to treat individual patients (1, 6). Because of their antiresorptive properties, bisphosphonates are the medical treatment of choice in symptomatic cases similar to conditions associated with bone resorption (5, 7, 8). Though pamidronate and alendronate are commonly used in various studies for the treatment of FD (9–11), the literature on zoledronic acid use in FD is limited (11–15). Compared to pamidronate, zoledronic acid requires fewer doses leading to better compliance with minimal side effects (6, 16). It has even shown promising results in cases of FD unresponsive to pamidronate therapy. We report the clinical and radiological response of zoledronic acid in children and adolescent with FD.

## Materials and Methods

Between April 2014 and March 2019, 12 children with FD were treated with the zoledronic acid infusion. The medical records of these patients were reviewed retrospectively, including the clinical features, radiological findings, and treatment. The patients with at least 2-year follow-up and having all clinical and radiological details were included in the study to look for the clinical, radiological and biochemical response of zoledronic acid treatment. Our institutional ethics review committee (IEC) waived the study (IEC waiver No: T/IM-MF/Ortho/19/63). All parents were counseled regarding intravenous zoledronic acid therapy, and it was administered after their consent. A total of 10 patients were included in the study, as 2 patients were excluded because of inadequate follow-up.

Before the commencement of the therapy, all patients underwent a radiographic study with baseline hemogram, liver function test, and renal function test. The biochemical assay of serum calcium, phosphorus, and ALP was performed using Beckman Coulter (AU5800 Clinical Chemistry Analyzer, Danaher Corporation, CA, USA). Serum vitamin-D3 and parathyroid hormone were estimated using electrochemiluminescence method. The clinical examination included pain, deformity, and spontaneous fracture. Pain score was evaluated by the visual analog scale (VAS) in children above 7 years (*n* = 6). It was rated on a scale from 0 to 9: 0 for no pain, 1–3 for low, 4–6 for moderate, and 7–9 for severe. Core needle biopsy and three-phase bone scan (^99m^ Tc-MDP) were performed in all cases to confirm the diagnosis and to know all sites of involvement in the body.

The children received intravenous zoledronic acid (Zometa, Panacea Pharmaceuticals) at a dose of 0.1 mg/kg as an infusion over 1 h immediately after diagnosis of fibrous dysplasia. Calcium and vitamin D supplementations were given to all patients. Associated fractures of the long bones were treated using plaster cast or fixation as appropriate. One patient with a huge osteolytic lesion in the tibia and pathological fracture underwent excision and bone transport. The patients were followed up at 6 weeks, 3 months, 6 months, 12 months, and at every 6-months interval subsequently. At each follow-up, they were evaluated clinically (pain and new onset of spontaneous fracture) and radiologically (anteroposterior and lateral views). If the VAS score was <3, and there was no new fracture, the treatment response was considered as positive. The radiological improvement, as evaluated by an experienced musculoskeletal radiologist was based on cortical thickening (>2 mm), progressive ossification of the lesion, and decrease in the diameter of the lesion (>20% of decrease). This radiological response was evaluated from the X-rays done at the 6 monthly interval. If the radiological improvement was consistent and progressive at every subsequent follow-up, it was considered as a positive response to the treatment. Normalization of ALP was considered as the biochemical marker of lesion healing. Severely symptomatic children (VAS > 3, impending fracture or new-onset fracture) without radiological improvement even after normalization of ALP were continued with zoledronic acid treatment. The zoledronic acid infusion was repeated at an interval of 6 months until 2 years. Then the drug was stopped for 1 year and again re-started depending on the clinical, radiological, and biochemical response. If there was acceptable clinical (VAS < 3, no new fracture), radiological (normal cortical thickness, lesion healed >2/3 of the diameter with complete or near-complete ossification) and biochemical improvement (normalization of ALP), then the treatment was stopped. The mean follow-up period was 32.6 months (range, 24–60 months). Statistical analysis was performed using the SPSS software (version 20). Descriptive statistics were expressed as number, percentages, mean ± SD or median as appropriate. Differences between the pretreatment and posttreatment parametric values were assessed by paired *t*-test. *P* < 0.05 was considered statistically significant.

## Results

### Baseline Characteristics

The average age of presentation was 9.1 years (range, 4–17 years). There were four male and six female patients. Based on radiological findings, there were eight PFD and two MFD. All patients had lower limb involvement with complain of pain, deformity, swelling, tenderness, or pathological fracture (*n* = 4). Bone biopsy using core needle confirmed the diagnosis in all cases. Three patients with PFD had associate systemic features like a café-au-lait spot or precocious puberty.

### Response to Zoledronic Acid

**Figure 1:**
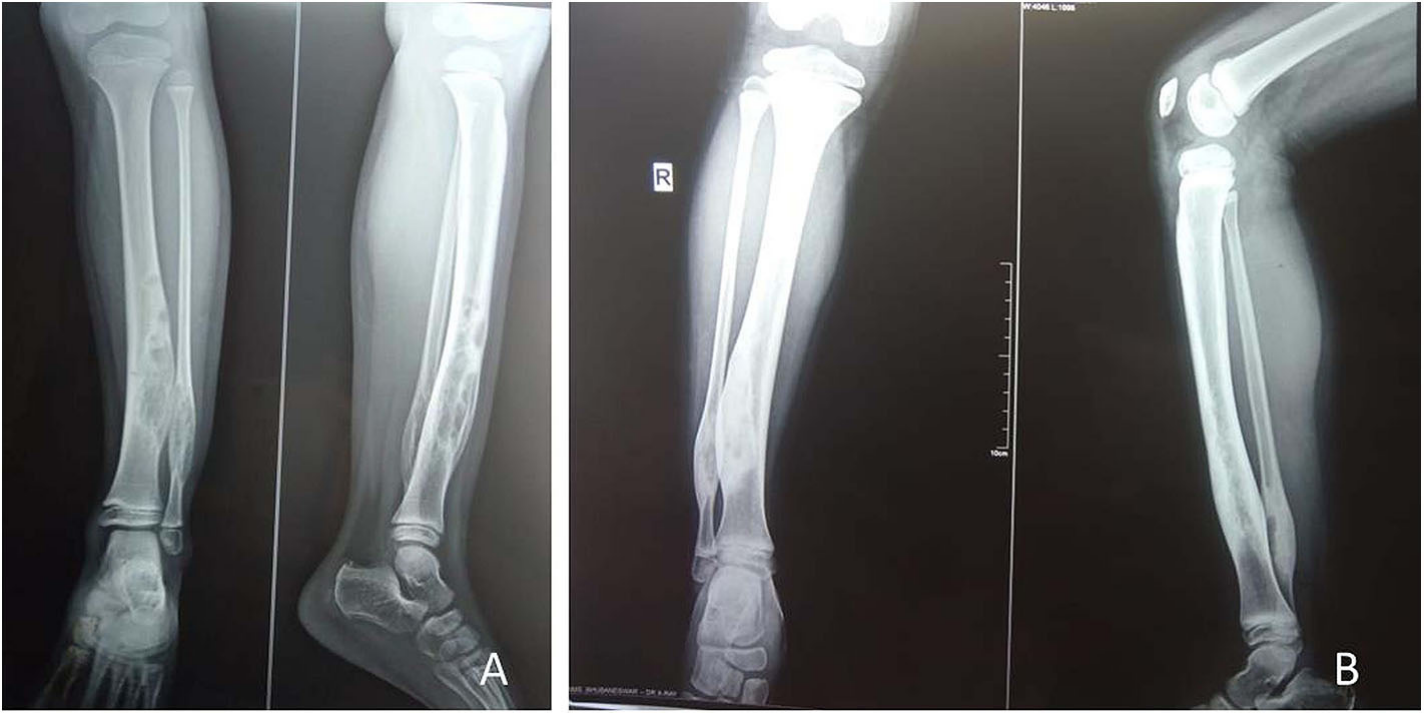
A 5-year-old female child with polyostotic fibrous dysplasia (PFD) of tibia, fibula **(A)**; follow up radiograph at 6 months revealed complete healing of the lesion **(B)**.

**Figure 2:**
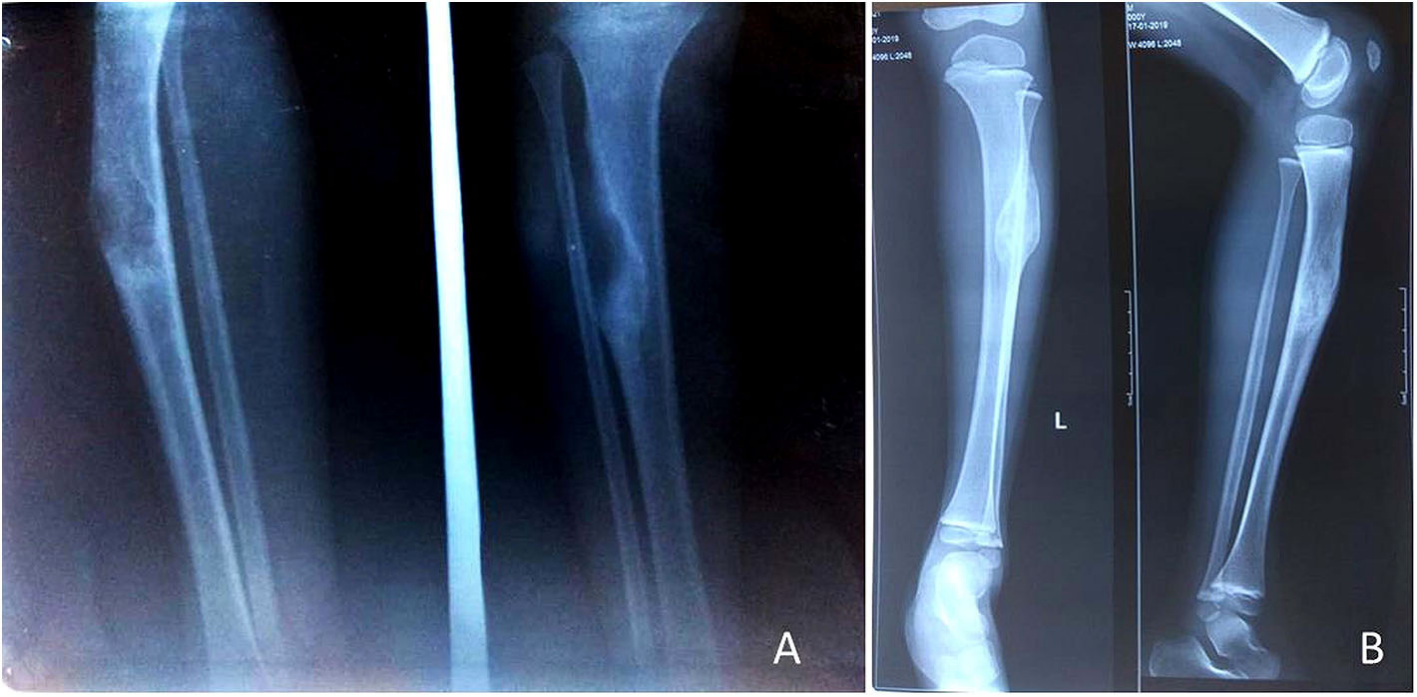
A 5-year-old male child with mono-ostotic fibrous dysplasia (MFD) of proximal tibia **(A)**. The lesion completely healed at3 months after zoledronic acid infusion **(B)**.

**Figure 3:**
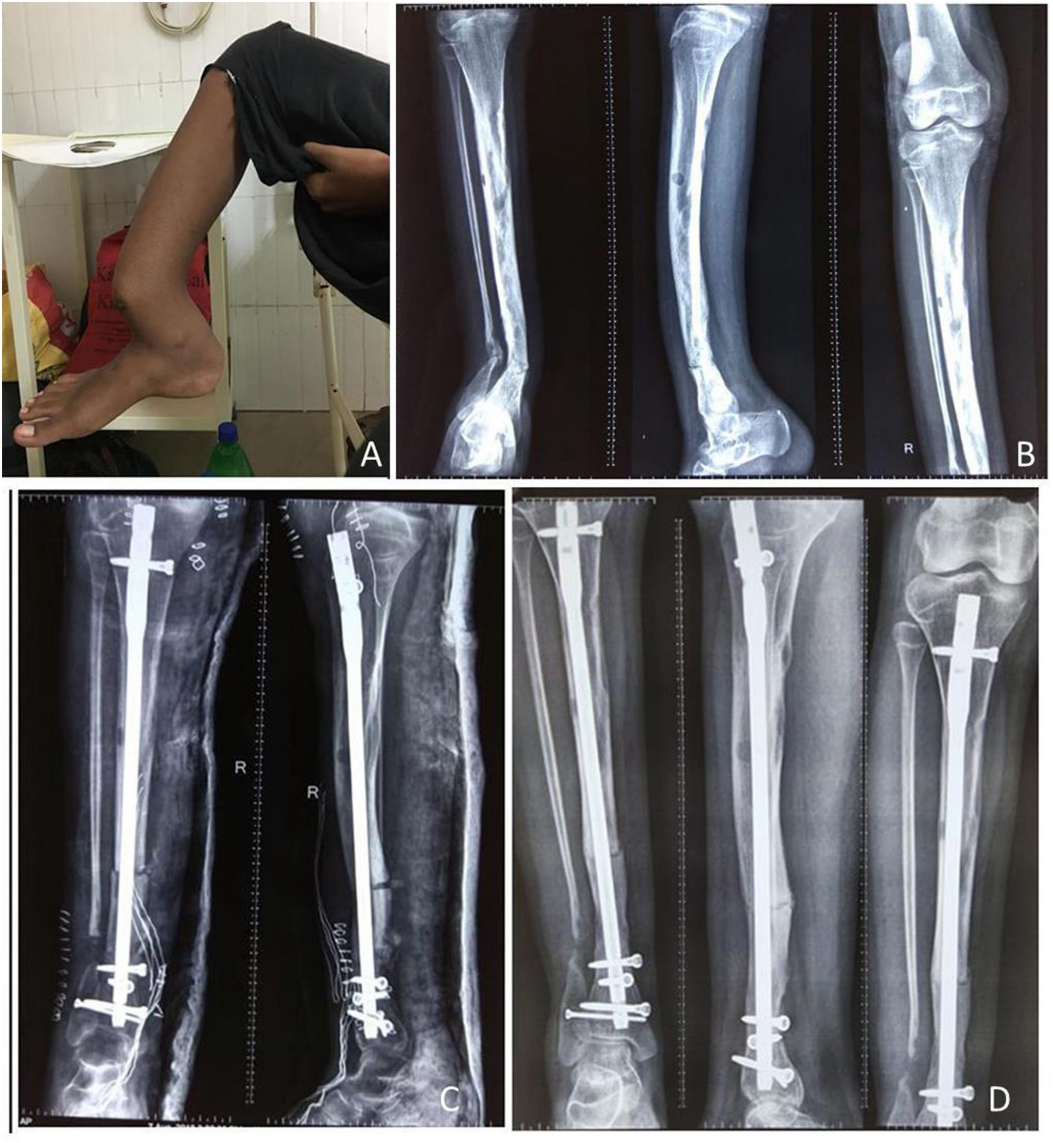
A 17-year-old adolescent girl presented with PFD involving the right tibial shaft and fibula **(A,B)**. After 2 months of zoledronic acid infusion, corrective osteotomy and intramedullary nailing was performed **(C)**, follow-up radiograph at 6 months shows complete healing of the lesion **(D)**.

**Figure 4:**
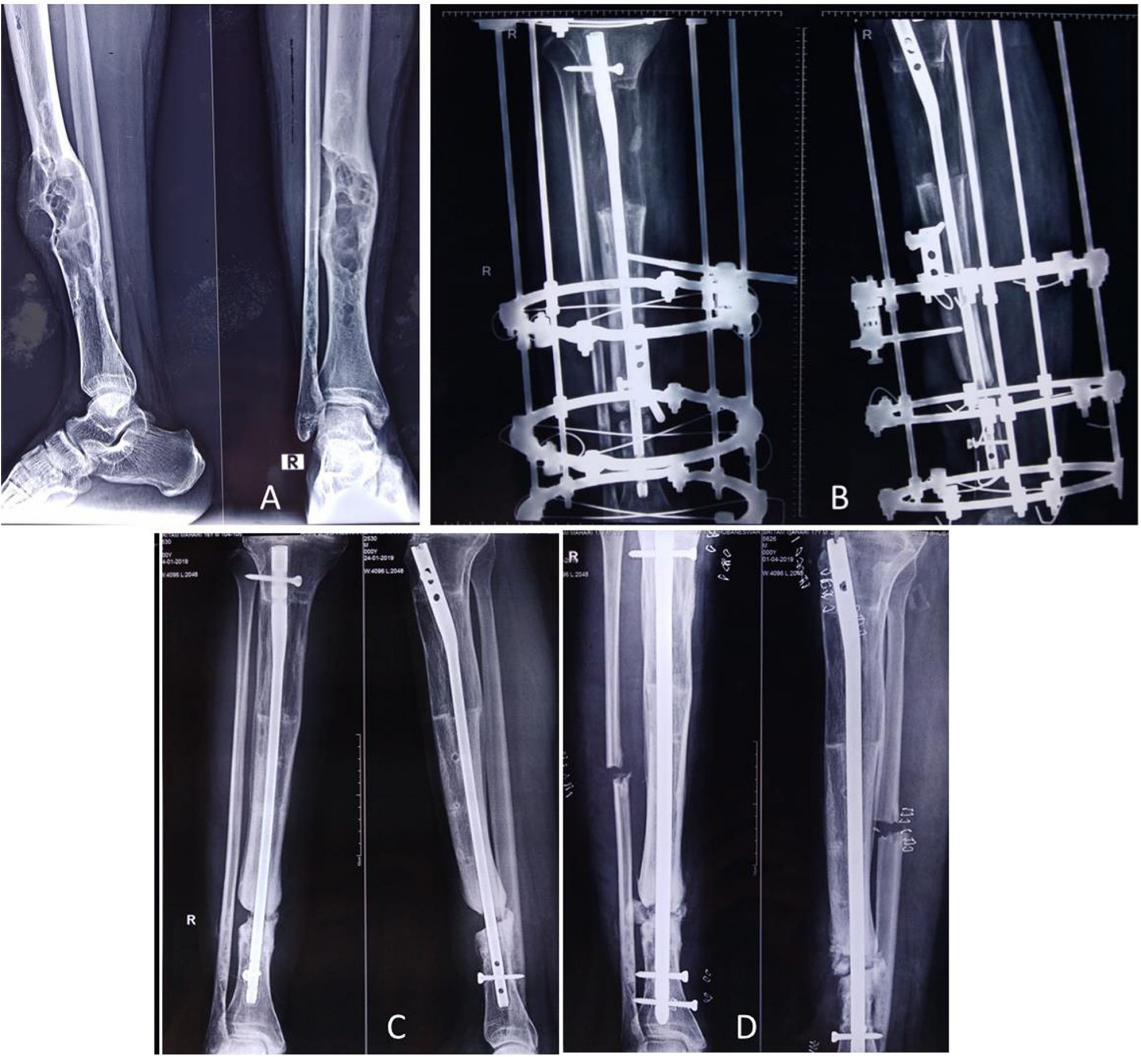
A 17-year-old male presented with PFD involving the right tibial shaft and fibula with an inability to walk **(A)**. Zoledronic acid was infused, and then complete excision of the lesion (of 12 cm length) with bone transport was performed over an intramedullary nail **(B)**; desired bone transport was completed after 6 months. After subsequent consolidation of the transported bone, the patient was allowed to walk. He presented after 1.5 years with nonunion at the distal tibia **(C)**, and it was bone grafted **(D)**. The lesion healed completely.

**Figure 5:**
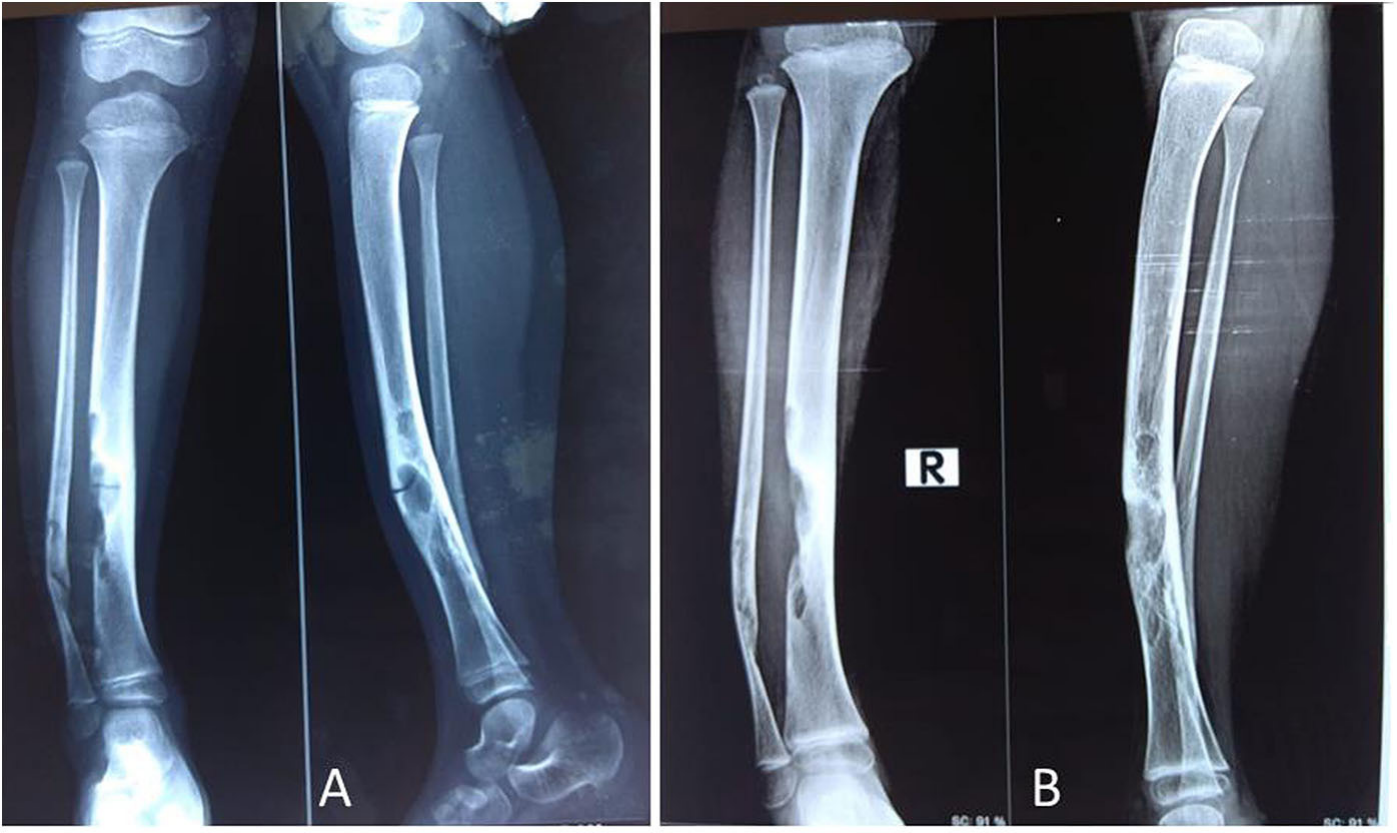
A 4-year-old female child presented with PFD involving tibia and fibula with pathological fracture **(A)**; the fracture got united, and the lesion healed up within 3 months of IV zoledronic acid infusion and plaster casting **(B)**.

**Figure 6:**
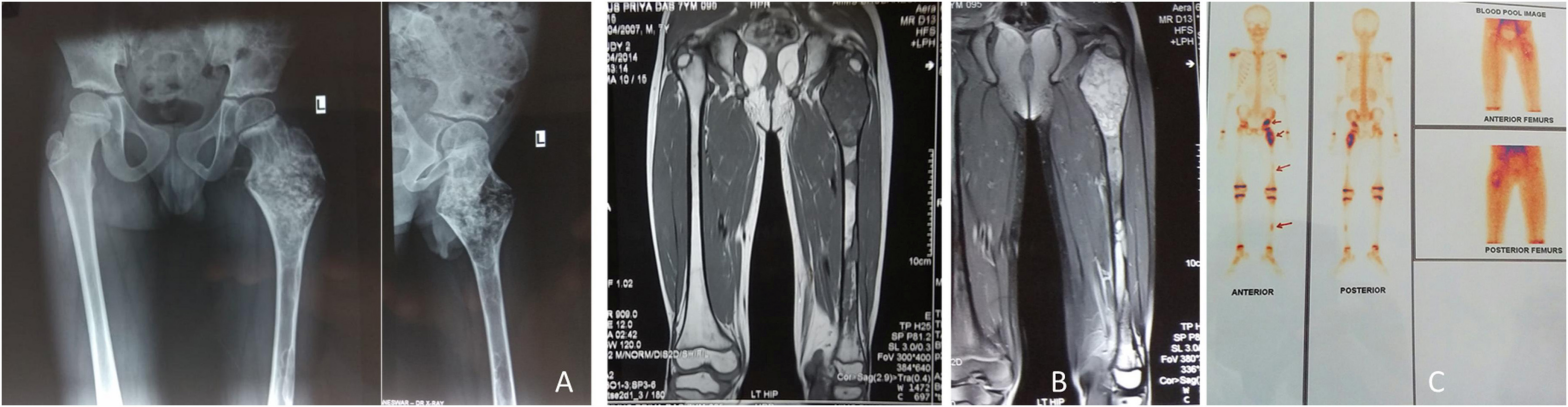
A 7-year-old male child presented with (PFD) involving left proximal femur, femoral shaft, left tibia and pelvis **(A)**. MRI showed T2W hyperintense signal, and T1W hypointense signal **(B)**. Three-phase bone scan revealed increased uptake at the involved sites **(C)**.

**Figure 7:**
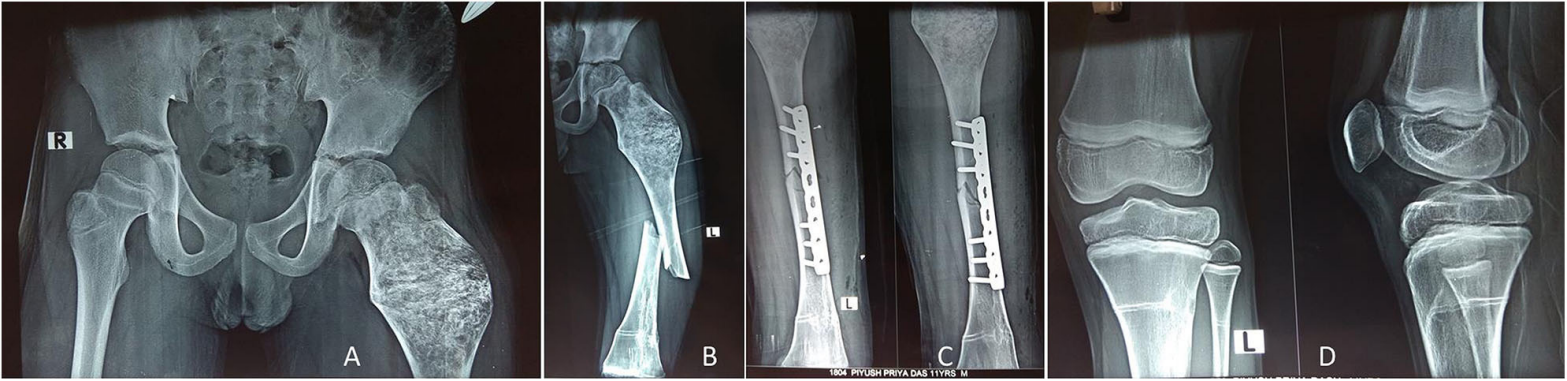
The child in Figure 6 received IV zoledronic acid infusion at every 6-month interval until 2 years, and the lesion healed up with mineralization **(A)**. Four years later, the child presented with femoral shaft fracture (but not in the lesion area) after a fall while playing football **(B)**, and it was treated with open reduction internal fixation with a plate **(C)**. The X-ray around the knee showed multiple Harris growth arrest lines (zebra stripe sign) **(D)**.

All patients received intravenous zoledronic acid (no other bisphosphonates before or during therapy), and on an average, each of the patients received 2.8 doses of zoledronic acid (range, 1–5 doses). Compliance was good in all the patients. We observed a significant improvement in the radiograph of the bony lesions with evidence of filling up of the osteolytic lesions and cortical thickening in all patients (median duration of healing 20 months). However, the deformity persisted in the non-surgical group (Table 1, Figures 1–7). The fracture united within 3 months of treatment (median healing time 2.1 ± 0.6 months) (Figure 3). There was no incidence of new fracture in the lesion sites after treatment with zoledronic acid. There was a significant reduction of pain in the affected bone (VAS decreased from 8 to 2.3). The VAS was <3 in all these children, which was considered as a significant improvement. All the patients reported improvement in activity levels. All patients were self-ambulatory without any support (manual muscle power testing, MRC 5/5 around the hip, knee, and ankle). They all resumed their normal activities of playing and going to school.

**Table 1:** Demographic and treatment details of children with fibrous dysplasia (FD).

**Patients**	**Age/sex**	**Clinical features**	**Radiology**	**Other system features**	**Treatment**	**Follow-up**	**Outcome**	**Complications**
1	7/M	Left hip pain and difficulty in walking since past 2 years	X-ray: A large osteolytic lesion in the proximal femur with widening Bone scan: Multiple site uptake including Left proximal femur, femoral shaft, tibia, and pelvis	-	Inj. zoledronic acid infusion (5doses)	60 months	Reduction in pain, increased activity levels. Radiological evidence of filling up of the bone defect	Transient fever, myalgia, flu symptoms after zoledronic acid infusion. Multiple physeal growth arrest lines around periarticular bones of the knee visible after 4 years
2	4/F	Swelling, pain, and deformity of the right leg following a fall while playing, history of bowing of Lt leg−6 months	Pathological fracture with the osteolytic lesions in the right tibia and fibula		Plaster casting was done along with injection zoledronic acid-1 dose	30 months	Fracture got united, and the lesion healed up at 3 months. The child is pain free and actively mobile	Transient fever and myalgia after zoledronic acid infusion
3	17/F	Progressive bowing deformity, pain in the right leg, and inability to bear weight since 1 year	Multiple lytic lesions in Rt tibial shaft, and fibula, fracture tibial shaft. Bone scan: distal femur, tibia, fibula		Inj. zoledronic acid infusion 3 doses. Deformity correction with osteotomy and intramedullary nailing of the right tibia	28 months	Fracture got united, and the lesion was filled up. The girl is walking normally with no residual deformity or pain	Transient fever, bone pain, and myalgia after zoledronic acid infusion
4	5/M	Pain, swelling Lt tibia−3 months	Lytic lesion Lt tibia		Inj. zoledronic acid infusion one dose	24 months	Symptom-free with minimal residual swelling. Complete radiological healing	Transient fever, myalgia
5	13/M	Pain left leg−2 months	Lytic lesion proximal tibia		Inj. zoledronic acid infusion one dose	24 months	Complete healing of the lesion	Transient fever, myalgia, joint pain
6	9/F	Pain and fracture of long bones treated outside as osteogenesis imperfecta	Bone scan: skull bones, bilateral humeri, bilateral forearm bones, multiple ribs, thoracolumbar vertebrae, pelvic bones, bilateral femora and tibiae	Café-au-lait spots, hyperthyroidism	Fracture fixation of the femur with plate. Inj zoledronic acid infusion five doses	48 months	Decrease bone pain; osteolytic lesion healed up, need corrective osteotomy for deformed femur	Transient fever and flu-like symptoms. Multiple physeal growth arrest lines
7	8/F	Pain, swelling Lt leg−6 months, Fracture Lt tibia	Bone scan: Lt proximal femur, distal tibia, fibula		Inj. zoledronic acid 4 mg IV infusion three doses	24 months	No pain, the swelling subsided completely, the fracture healed, and there was an increased cortical thickness	Transient fever, myalgia, multiple physeal growth arrest lines
8	5 /F	Progressive deformity and pain Rt leg−2 years	Lytic lesions in the shaft and distal part of Rt tibia and fibula. Bone scan revealed additional uptake in Rt proximal femur as well	Café-au-lait spots, No endocrinopathy	Inj. zoledronic acid IV infusion-4 doses	28 months	A marked decrease in pain, osteolytic lesion healed up	Symptomatic hypocalcaemia that was transient and treated with injection calcium gluconate. Transient fever, myalgia, multiple physeal growth arrest lines
9	6/F	Painful limp with 2 cm shortening−3 months	Multiple osteolytic lesions in the pelvis and left femur	Precocious puberty, increased serum estrogen level	Inj. zoledronic acid IV infusion-4 doses	24 months	A marked decrease in pain, osteolytic lesion healed up	Transient fever, myalgia, multiple physeal growth arrest lines
10	17/M	Pain and swelling in the right tibia with the inability to bear weight. The swelling was producing egg-shell crackling sound on finger pressure, pathological fracture	Multiple osteolytic lesions in right tibia and fibula		Inj. zoledronic acid IV infusion followed by tumor excision (12 cm) and bone transport over a nail. Total one dose of zoledronic acid	36 months	The fracture got united after bone grafting, and the lesion completely healed up	Transient fever, myalgia

*M, male; F, female; Inj., injection*.

All patients were having normal serum calcium and phosphorous level at the time of admission. Serum ALP was elevated in all but two patients before treatment. With treatment, the serum ALP significantly decreased (*p* = 0.04, Table 2). The serum vitamin D level was normal in three patients, and there were associate hypovitaminosis D in the remaining seven patients. These patients were treated with cholecalciferol supplementation (one dose of 600,000 units of vitamin D orally followed by a maintenance dose of 5,000 IU/day for 3 months) prior to the zoledronic acid infusion.

**Table 2:** Changes in biochemical parameters with zoledronic acid treatment in FD.

**Parameters**	**Mean (±SD) before treatment**	**Mean (±SD) on treatment (12 months)**	***P*-value**
Serum calcium (mg/dl)	8.67 (0.97)	8.42 (0.56)	0.44
Serum phosphorus (mg/dl)	4.22 (0.41)	4.38 (0.56)	0.22
Serum alkaline phosphatase (IU/L)	895.75 (79.64)	544.12 (47.35)	0.04^*^

* *Significant, paired t-test*.

### Side Effects

All patients developed transient fever and myalgia for 2–3 days immediately after the administration of zoledronic acid infusion. One child had symptomatic hypocalcemia, and she was effectively treated with calcium gluconate injection. Multiple physeal growth arrest lines were visible in five patients (Figure 7D).

## Discussion

Zoledronic acid is a potent third-generation bisphosphonate that markedly decreases osteoclast-mediated bone resorption (16). Nevertheless, its efficacy and safety have never been compared to other bisphosphonates in children with FD. One to four cycles of intravenous zoledronic acid in pediatric FD have shown to relieve pain, decreased the markers of bone turnover, and improved radiologic findings (6, 11–19). In agreement with these studies, our observation revealed that there were significant improvements in pain and radiological findings. The associated pathological fractures healed in all patients, and there was no new fracture. The decrease in serum ALP (as a marker of bone turnover) after treatment confirmed the effectiveness of zoledronic acid infusion in children with FD (15).

The use of bisphosphonates in FD is still controversial (10, 20–22). While many studies report that it as a safe and effective medical treatment, a few studies do not show its usefulness (9, 20, 23, 24). The use of bisphosphonates in FD is based on the fact that there is increased osteoclastic activity in the bony lesions (9–15). Boyce et al. in their randomized controlled trial (RCT) concluded that alendronate was responsible for the reduction of bone resorption markers and improvement in areal bone mineral density without any significant reduction in pain and functional parameters (9). The authors did not recommend alendronate treatment in FD. Compared with oral therapy, maximal suppression of bone resorption markers occurs more rapidly after intravenous infusion, which bypasses the GI tract and thus reduces the risk of adverse effects related to oral bisphosphonates. Lala et al. reported the outcome of intravenous pamidronate in nine children with McCune–Albright syndrome (21); in the treatment period of 0.5–3.5 years, no spontaneous fracture occurred. Bone pain and gait abnormality disappeared after two to three therapeutic cycles. However, the cranial asymmetry and limb length discrepancy remained unchanged. The bone turnover markers (serum ALP and urine hydroxyproline levels) reduced after treatment indicating the drug activity at the lesional level. However, it is difficult to comment whether the reduction of these markers are only because of suppression of resorption inside the lesion or in the normal bone as an increased bone mineral density was also observed in non-lesional sites (24). However, the author did not observe radiographic and scintigraphic evidence of lesion healing in these children as seen in adults. The authors concluded that pamidronate is an effective and safe treatment for FD in children and adolescents with McCune–Albright syndrome. A similar observation was reported by Plotkin and his associates, who observed improvement in pain and bone turn over markers in children and adolescents. However, there was no radiographic evidence of the filling of lytic lesions or thickening of the bone cortex surrounding the lesions in any patient (22).

Compared to pamidronate, zoledronic acid is more potent with fewer adverse reactions. It has an inherent antimetastatic role in malignant bone tumors, and it has shown promising results in FD unresponsive to pamidronate therapy. Because of its less frequent administration (6 monthly or yearly), patient compliance is better. Wu et al. treated a case of PFD in a 21-year old male with 5 mg IV zoledronic acid/year for 4 years (12). They noted significant improvement in pain, filling of the bone defect and significant decrease in both serum collagen type 1 cross-linked C-telopeptide and type 1 procollagen N-terminal (P1NP) from extreme high baseline levels. They observed an unexpected direct increase in P1NP after the fourth infusion, possibly indicating that zoledronic acid antiresorption effect was approaching the maximum, and a sign to discontinue the treatment. However, Ganda et al. did not observe the same improvement in their patient after zoledronic acid infusion (25). They started zoledronic acid in the fifth decade when the disease was more severe and extensive with deformity. It seems the drug is effective when administered early. Probably, it is one of the reasons for its effectiveness, particularly in children and adolescent. Wang et al. noted a decline in ALP by 30.3% of baseline in the patient receiving alendronate (*n* = 1) vs. 22.7% in the pamidronate group (*n* = 10) and 34.1% in the zoledronic acid group (*n* = 11). Sixty-four percent of patients had relief from bone pain. They advocated that bisphosphonate treatment is safe and cause no apparent impairment in children's linear growth (15). Bhadada et al. (26) also noted significant improvement in pain and healing of FD lesion after bisphosphonate treatment in seven patients with pathological fracture. The associated deformities in FD needed surgical correction.

Regarding the endpoint of treatment, most of the authors use bone turn over markers as the surrogate endpoint. However, the bone turnover marker facilities were not available in our institute, and we just followed up our patients clinically, radiographically, and by measuring serum ALP. As per current recommendation, the bone turnover markers (N-telopeptide and ALP) should be monitored at 6-monthly intervals and bone mineral density every year during treatment to assess the efficacy of bisphosphonate therapy.

In an interesting article published recently, Florenzano et al. reported that the bone turn over markers that decreased after bisphosphonate treatment in FD is actually a reflection of an age-dependent declining trend and not because of bisphosphonate treatment. They did not observe any correlation between the pain in FD and the bone turnover markers. So, they concluded that bisphosphonate treatment does not significantly impact the age-dependent decrease in bone turnover, nor does it prevent the progression of FD disease burden in children. They caution about the use of bisphosphonate in FD because of the side effects, and they also questioned on those studies where bone turnover markers were used as surrogate endpoints (21). Plotkin et al. did not observe any difference in histomorphometric findings in FD tissue of patients receiving pamidronate and untreated patients (22). In another case report, Corsi and colleagues noted radiographic and histologic evidence of bisphosphonate exposure only in normal bone but not within the FD lesion of a child (27). Thus, many authors hypothesized that probably the decrease in bone turn over markers observed with bisphosphonate treatment in subjects with FD is a combination of the effect of the drug on healthy bone and the age-related decrease in FD activity. To avoid the controversy of bisphosphonate whether it really decreases the markers or because of the normal aging process, a very recent article by Guerin Lemaire et al. (28) used serum periostin level as a biochemical marker to assess the severity and to monitor the treatment in FD. They measured the serum periostin level in healthy individuals who were the control group. They observed a significantly lower level of serum periostin level in patients who received bisphosphonate compared to untreated patients (mean = 953 vs. 1,370 pmol/l, *p* = 0.002). This study also proved that intravenous zoledronic acid is effective in FD. Early treatment may be more effective, and hence, children do respond very well with minimal adverse reactions to the drug.

Although transient adverse events such as flu-like symptoms were seen in all cases after zoledronic acid infusion, long-term use side effects such as iatrogenic fracture and jaw necrosis were not seen (29). Five patients developed multiple physeal growth arrest lines warranting a regular follow-up with radiographs. The zebra stripe sign is the radiological manifestation of cyclical bisphosphonate therapy before the physeal closure and is observed in about 50% of users (30). These sclerotic lines are developed because of temporary interruption of growth plate cartilage resorption induced by the bisphosphonates and the relative increase in bone formation with high levels of osteoblastic activity on the metaphyseal side of the growth plates. However, these sclerotic lines are transient and disappear after discontinuation of bisphosphonate. Except in one patient (who had transient symptomatic hypocalcemia), there was no report of any adverse effects in our series. We used a higher dose of zoledronic acid (0.1 mg/kg) based on our previous experience that explains the increased incidence of flu-like symptoms and hypocalcemia. The side effects of the zoledronic acid can be minimized with lower effective dose (0.025 mg/kg) (31).

There are certain limitations in our observations. First, being a retrospective study, biases and loss of some data are unavoidable. The number of patients recruited in this study is small. FD is an uncommon bony disorder, and there is not enough literature on its treatment. Even a few cases on this disease are a substantial contribution to the literature. Second, bone pain and limitation of movement could not be evaluated by validated quantitative tools in all the patients in a standardized manner that may affect the clinical evaluation. Third, the placebo effect of the injection could not be excluded from being a single-arm study. Fourth, as we had no facility of measuring the bone turnover markers at our institution, the decision to stop medication was based on clinical improvement, radiological improvement, and ALP level. However, there are no definite guidelines to decide the endpoint of treatment as the bone mineral turnover markers do not correlate to the disease activity; sometimes the radiological improvement is not associated with a decrease in biochemical markers (9, 14). Finally, the study group was heterogeneous as we included both mono-ostotic and polyostotic fibrous dysplasia with three children of McCune–Albright syndrome. Zoledronic acid acts on the bony lesions, and there have been no reports of effects of this drug on other systemic features of FD. Hence, we believe that the study heterogeneity has no impact on our interpretation of the drug in FD.

In conclusion, the clinical and radiological improvement of fibrous dysplasia after intravenous zoledronic acid infusion in children is promising. It is well-tolerated, and there is good compliance because of the 6 monthly treatment. However, we need further randomized control trials with a large sample size and longer follow-up to establish Zoledronic acid as a first-line medical treatment in FD.

## Data Availability Statement

All datasets generated for this study are included in the article/supplementary material.

## Ethics Statement

The studies involving human participants were reviewed and approved by Institutional ethics Committee, AIIMS Bhubaneswar. Written informed consent to participate in this study was provided by the participants' legal guardian/next of kin. Written informed consent was obtained from the minor(s)' legal guardian/next of kin for the publication of any potentially identifiable images or data included in this article.

## Author Contributions

The initial concept and draft was prepared by ST, RD, and AS. Data was collected by SS, SV, DP, and KA. The final draft was prepared by ST, RD, and DP. Intellectual contents were provided by RD, KA, and AS. All authors read the final manuscript and approved it for submission.

## Conflict of Interest

The authors declare that the research was conducted in the absence of any commercial or financial relationships that could be construed as a potential conflict of interest.
